# Longitudinal comparison of quality of life in patients undergoing laparoscopic Toupet fundoplication versus magnetic sphincter augmentation: Observational cohort study with propensity score analysis

**DOI:** 10.1097/MD.0000000000004366

**Published:** 2016-07-29

**Authors:** Emanuele Asti, Gianluca Bonitta, Andrea Lovece, Veronica Lazzari, Luigi Bonavina

**Affiliations:** Department of Biomedical Sciences for Health, Division of General Surgery, University of Milan Medical School, IRCCS Policlinico San Donato, San Donato Milanese, Milan, Italy.

**Keywords:** gastroesophageal reflux, GERD-HRQL score, laparoscopic Nissen fundoplication, laparoscopic Toupet fundoplication, lower esophageal sphincter, magnetic LES augmentation, propensity score

## Abstract

Only a minority of patients with gastro-esophageal reflux disease (GERD) are offered a surgical option. This is mostly due to the fear of potential side effects, the variable success rate, and the extreme alteration of gastric anatomy with the current gold standard, the laparoscopic Nissen fundoplication. It has been reported that laparoscopic Toupet fundoplication (LTF) and laparoscopic sphincter augmentation using a magnetic device (LINX) can treat reflux more physiologically and with a lower incidence of side-effects and reoperation rate. We present the first comparing quality of life in patients undergoing LTF versus LINX.

Observational cohort study. Consecutive patients undergoing LTF or LINX over the same time period were compared by using the propensity score full matching method and generalized estimating equation. Criteria of exclusion were >3 cm hiatal hernia, grade C–D esophagitis, ineffective esophageal motility, body mass index >35, and previous upper abdominal surgery. The primary study outcome was quality of life measured with the Gastro-Esophageal Reflux Disease-Health Related Quality of Life (GERD-HRQL) questionnaire. Secondary outcomes were proton pump inhibitors (PPI) use, presence of gas-related symptoms or dysphagia, and reoperation-free probability.

Between March 2007 and July 2014, 238 patients with GERD met the criteria of inclusion in the study. Of these, 103 underwent an LTF and 135 a LINX procedure. All patients had a minimum 1-year follow-up. Over time, patients in both groups had similar GERD-HRQL scores (odds ratio [OR] 1.04, confidence interval [CI] 0.89–1.27; *P* = 0.578), PPI use (OR 1.18, CI 0.81–1.70; *P* = 0.388), gas-related symptoms (OR 0.69, CI 0.21–2.28; *P* = 0.542), dysphagia (OR 0.62, CI 0.26–1.30; *P* = 0.241), and reoperation-free probability (stratified log-rank test = 0.556).

In 2 concurrent cohorts of patients with early stage GERD undergoing LTF or LINX and matched by propensity score analysis, health-related quality of life significantly improved and GERD-HRQL scores had a similar decreasing trend over time up to 7 years of follow-up. We conclude that LTF and LINX provide similar disease-specific quality of life over time in patients with early stage GERD.

## 1 Introduction

Gastro-esophageal reflux disease (GERD) is a highly prevalent disease affecting up to 30% of the population in Western countries. The burden of the disease in the general population is probably underestimated because many people with symptoms do not consult a physician. The diagnosis of GERD has increased more than 200% from 1998 to 2005, and it is now the most common reason for access to gastroenterology outpatient clinics.^[1]^ The impact of GERD on quality of life is worse than other common chronic conditions such as diabetes, arthritis, and congestive heart failure. Gastroesophageal reflux interferes with physical activity and social functioning, disturbs sleep, reduces productivity at work, and leads to increased healthcare resource utilization; therefore, the primary goal of therapy in uncomplicated GERD is to improve patient's symptoms and quality of life over time.^[2]^

Proton pump inhibitors (PPI) represent the first-line therapy approach in GERD; however, nearly 40% of patients have inadequate symptom relief despite high dose medication. This is mainly because the therapeutic gain for the relief of regurgitation is modest and considerably lower than that for heartburn.^[3]^

Laparoscopic Nissen fundoplication is generally recognized as the gold standard of antireflux surgery worldwide. On the other hand, the Nissen procedure is highly operator-dependent, has a variable success rate, can lead to potential side effects, and is regarded by some a sort of overtreatment for patients with mild to moderate GERD. As a consequence, the number of Nissen fundoplications has steadily declined over the recent years.^[4]^

The debate about the choice of the most appropriate surgical technique to provide optimal reflux control while minimizing the side effects is still ongoing. It has been assumed that the laparoscopic Toupet fundoplication (LTF) would provide less outflow resistance, thereby lowering the dysphagia and the bloating rate, and some surgeons favor this operation arguing that a partial fundoplication is more physiological and effective at least in patients with “mild” disease.^[5]^

Over the past 15 years we have been performing LTF in patients with early stage GERD and in those with large hiatal hernia or ineffective esophageal motility. In 2007, we started to perform laparoscopic magnetic sphincter augmentation with the LINX device as part of a feasibility trial,^[6]^ and this is still an option we offer to patients with early stage GERD. It is a simple standardized laparoscopic procedure that does not alter gastric anatomy, provides relief of reflux-related symptoms without impeding the ability to belch or vomit, and is reversible if necessary.^[7]^ The LINX device is FDA approved and is currently available in the market.

The aim of this study was to assess and compare health-related quality of life over time in 2 concurrent cohorts of patients undergoing LTF or LINX matched by propensity score (PS).

## 2 Methods

### 2.1 Study design

Observational cohort study. All patients undergoing an LTF or LINX procedure between March 2007 and July 2014 were identified from a prospectively collected data base. The time frame was chosen to include patients undergoing both surgical operations during the same period and to allow for a minimum 1-year follow-up. Ethical approval was waived and written consent was not given to the patients because all data were analyzed anonymously. We adopted the STROBE criteria for reporting an observational study.^[8]^

Inclusion criteria were age >18 years, chronic GERD symptoms despite PPI use for at least 6 months, objective evidence of reflux at the pH study, and normal esophageal motility documented by manometry. Exclusion criteria were the following: hiatal hernia >3 cm, esophagitis grade C–D, ineffective esophageal motility, body mass index >35, and previous esophago-gastric surgery. The primary outcome was postoperative quality of life measured with the validated and disease-specific Gastro-Esophageal Reflux Disease-Health Related Quality of Life (GERD-HRQL) questionnaire.^[9]^ Questionnaires were administered in the outpatient clinic to all patients. Secondary outcomes were PPI use, presence of gas-related symptoms or dysphagia, and reoperation-free probability. Patients in both groups were evaluated at 3 to 12 months, and then every 12 months with the GERD-HRQL survey plus questions about PPI use, gas-related symptoms and dysphagia.

### 2.2 Surgical procedure

Both the LTF and LINX procedures were performed according to a standard institutional protocol. In the LTF procedure, the right and left diaphragmatic crus were dissected in order to create a large retroesophageal window and encircle the esophagus en bloc with the posterior vagus nerve. The vagal branch to the gallbladder was routinely preserved. After division of the phrenoesophageal ligament, the esophagus was pulled down and dissection carried out in the mediastinum in order to obtain a 4 cm tension-free intra-abdominal esophageal segment. A posterior hiatoplasty was routinely performed. The gastrophrenic ligament along with the proximal short gastric vessels was divided and the fundus was wrapped behind the esophagus. Four cardinal nonabsorbable stitches were placed to held the fundoplication in place by tailoring the fundus symmetrically on both sides and without tension. The 2 proximal stitches were also fixed to the diaphragm. Finally, both edges of the fundic wrap were sutured to the right and left side of the esophageal wall using nonabsorbable running sutures.

In the LINX procedure, the peritoneal reflection overlying the esophago-gastric junction was divided, and the mediastinal cavity was not routinely entered in an attempt to preserve the phrenoesophageal ligament. A posterior hiatoplasty was added at discretion of the surgeon only when the hiatus appeared to be markedly enlarged. The vagal branch to the gallbladder was routinely preserved. The posterior vagus nerve and the area corresponding to the Z line were identified and a tunnel was created between the posterior vagus nerve and the esophagus. A special sizing instrument was used to measure the circumference of the esophagus and an appropriately sized device was placed through the tunnel. The ends of the device were then secured anteriorly.

### 2.3 Statistical analysis

Continuous data are presented as median and interquartile range (IQR). Categorical variables are shown as numbers and percentages. Wilcoxon signed-rank, Wilcoxon matched pairs signed-rank, Fisher exact, or χ^2^ tests and conditional logistic regression were performed as appropriate. Confidence intervals (CIs) at 95% confidence level, 2-sided statistical test with type I error = 0.05, and q-value (q) for multiple test with false discovery rate = 0.05. All analyses were carried out using R software package version 3.2.2.^[10]^

To reduce the impact of treatment selection bias inherent to an observational study, we compared postoperative GERD-HRQL of patients with LTF and LINX using the PS matching method. The PS, defined as the conditional probability of assignment to a treatment given a vector of particular observed covariates, is designed to mimic some of the particular characteristics of a randomized clinical trial in the context of an observational study. As appropriate and with caution, PS analysis allows estimation of relative risk in binary outcomes.^[11]^

We computed a PS for individual patients with logistic regression using demographic and clinical variables,^[12]^ and evaluated the interaction among all preoperative covariates and square terms without time-dependent variables. The generalized additive model was used to check linear assumption in PS model. The 2 patient groups were then matched using the PS full-matching method.^[13]^ The balance of baseline covariates after matching was assessed using the standardized difference of mean,^[14]^ and the overlap degree of PS distribution. We also performed sensitivity analysis^[15]^ to assess possible hidden bias due to unobserved confounders. We analyzed correlated repeated measure of GERD-HRQL, PPI use, presence of gas-related symptoms, and dysphagia over time with generalized estimating equation (GEE) in PS full-matched dataset; we used sandwich estimator and autoregressive of order 1 working correlation matrix.^[16]^ In particular, to account for skewed and doubly bounded nature of GERD-HRQL scores, we performed linear transformation of GERD-HRQL values. Then, we specified mean and variance function of beta distribution with logit as mean function.^[17]^ The linear predictor for means includes preoperative transformed GERD-HRQL, treatment, time, and time–treatment interaction. GEE takes into account that the PS method allows for estimation of marginal treatment effect and the matched nature of data. For models selection we used quasi-likelihood information criterion. Wald test and Wald CI were computed in GEE analysis. We established a 1.5 clinical effect-size threshold for the odds ratio (OR), a value that is compatible with clinical experience and published indices.^[18]^

The Kaplan–Meier reoperation-free probability curves were estimated separately for LTF and LINX in the PS full-matched sample. Stratified log-rank test was used to compare the curves. Hazard ratio was estimated with univariate marginal survival model with robust standard errors in PS full-matched data set.^[14]^ Proportional hazard assumption was tested. Reference group was LTF for all models.

## 3 Results

All 238 patients were successfully treated via a laparoscopic approach. The duration of the surgical procedure was 87 minutes (IQR 28) in the LTF group and 42 minutes (IQR 34) in the LINX group (*P* < 0.001). One patient in the LINX group had a respiratory arrest within the 1st hour postoperatively and was successfully resuscitated without consequences. Postoperative morbidity consisted of atrial fibrillation (n = 1), urinary retention (n = 1), and bleeding from a trocar site (n = 1), all occurring in the LTF group.

### 3.1 Preoperative patient characteristics

The preoperative patients’ characteristics are reported in Table 1. There were statistically significant differences in 6 of the 19 covariates. Thus, as typically occurs in observational studies, there were systematic differences in preoperative characteristics between the 2 treatments.

**Table 1:**
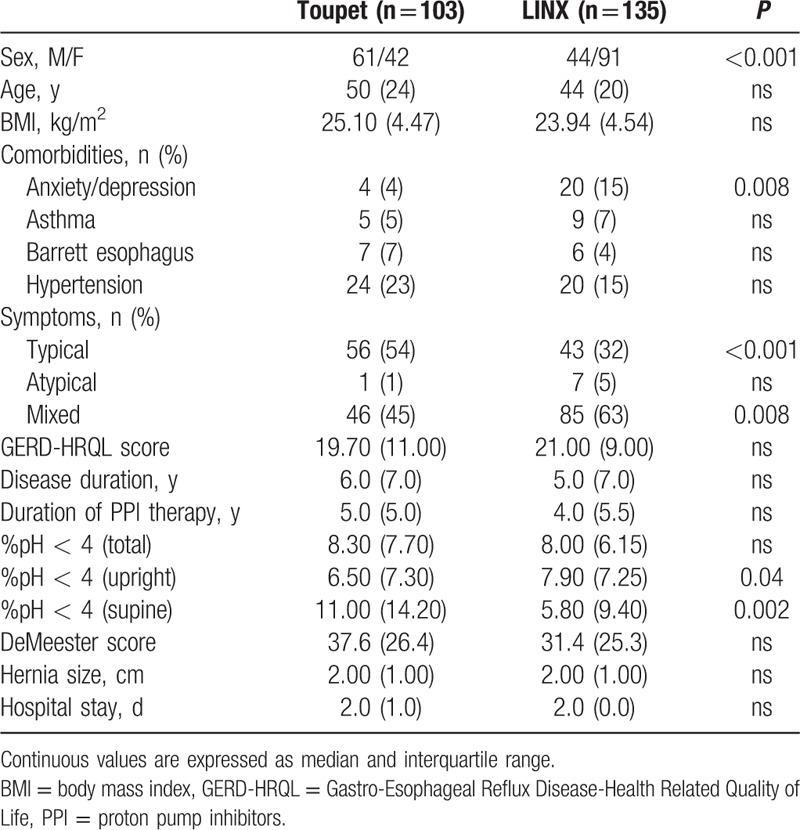
Baseline characteristics of the study sample.

In the PS full-matched data, 16 of the variables had absolute standardized differences of mean after matching that exceeded 0.10 (Table 2). The absolute standardized differences of mean ranged from 0.06 to 0.1, with a median of 0.044 (25th and 75th percentiles 0.023–0.071, respectively), indicating that the means and prevalences of continuous and dichotomous variables were very similar between treatment groups. The variance ratios for continuous variables ranged from 0.83 to 1.10, indicating that the variance variables were similar between the 2 treatment groups.

**Table 2:**
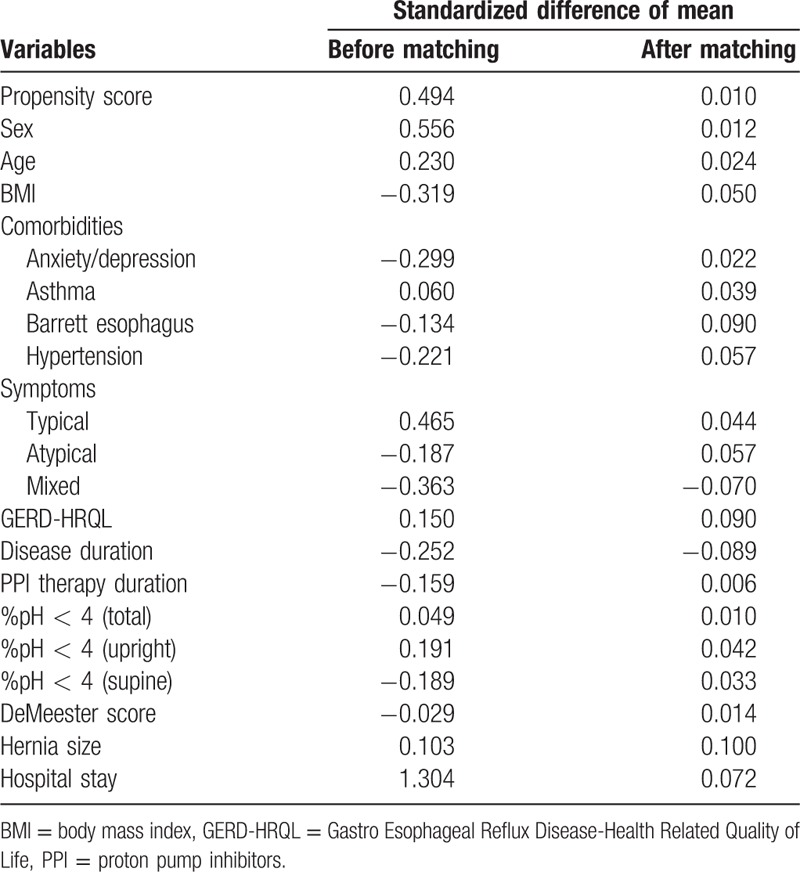
Standardized difference of mean before and after full propensity score matching with relative balance improvement (%) for each baseline characteristic.

### 3.2 Postoperative follow-up

All patients had a minimum 1-year follow-up. The mean postoperative follow-up was 42 and 44 months in LTF and LINX groups, respectively.

### 3.3 Quality of life

**Figure 1:**
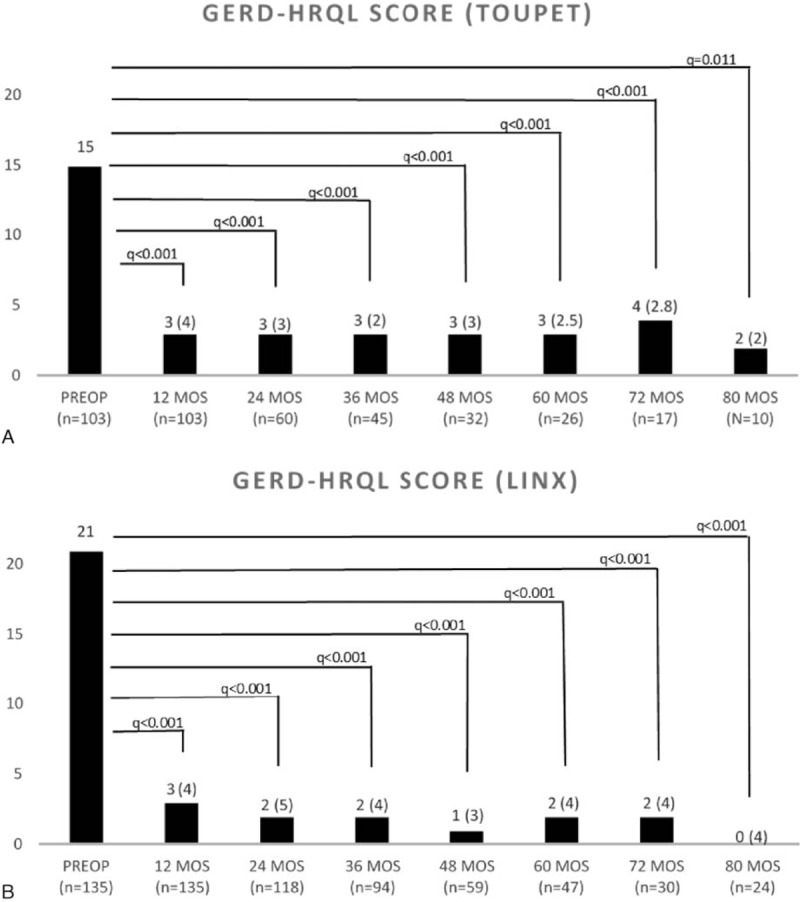
(A and B) Comparison of pre and postoperative GERD-HRQL scores in patients with LTF and LINX at each measured follow-up time. Wilcoxon matched pairs signed-rank, q values (q), and false discovery rate = 0.05 level. Values are expressed as median and interquartile range. GERD-HRQL = Gastro-Esophageal Reflux Disease-Health Related Quality of Life, LTF = laparoscopic Toupet fundoplication.

**Figure 2:**
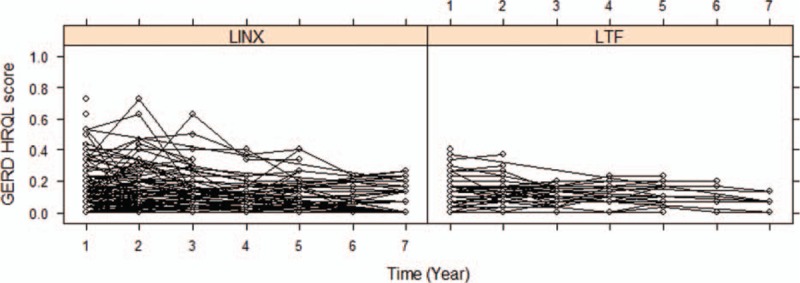
Spaghetti plot: trajectories over time of linearly transformed GERD-HRQL scores for each individual according to the surgical technique. GERD-HRQL = Gastro-Esophageal Reflux Disease-Health Related Quality of Life.

The GERD-HRQL score significantly decreased within normal values to a similar extent after both procedures (Fig. 1A and B). Parameters estimate by the beta GEE model for GERD-HRQL are shown in Table 3. Over time, there was no statistical difference in the GERD-HRQL scores between the LTF and LINX groups as indicated by the time–treatment interaction term in the GEE model (OR 1.04, CI 0.89–1.27; *P* = 0.578). Furthermore, the OR CI did not encompass the clinical significance previously established at 1.5 threshold. The raw analysis showed similar results (OR 0.99, CI 0.94–1.03; *P* = 0.528). Due to the fact that the pattern of change was the same over time in both groups, we assumed a linear trend and refitted the model by excluding the interaction term to investigate the trend of the logit of mean of GERD-HRQL. This showed a decreasing linear trend over time (GEE model time parameter −0.069, CI −0.104 to −0.032; *P* < 0.001) (Fig. 2).

**Table 3:**
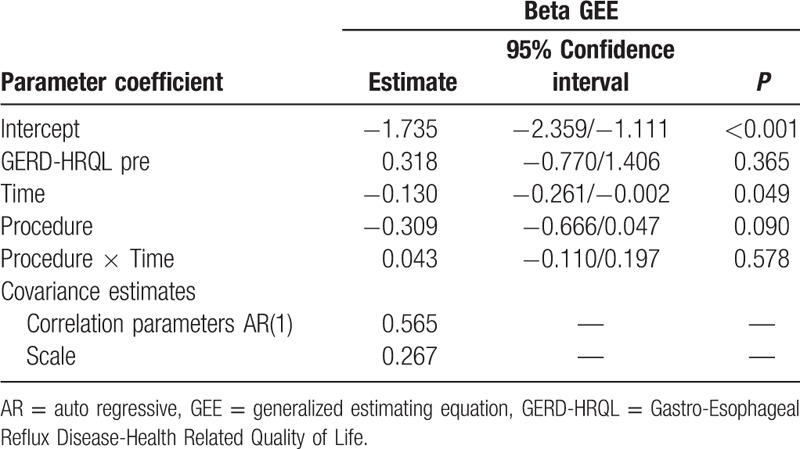
Results of beta GEE model for GERD-HRQL outcomes.

### 3.4 PPI use, gas-related symptoms, dysphagia, and reoperation rate

**Figure 3:**
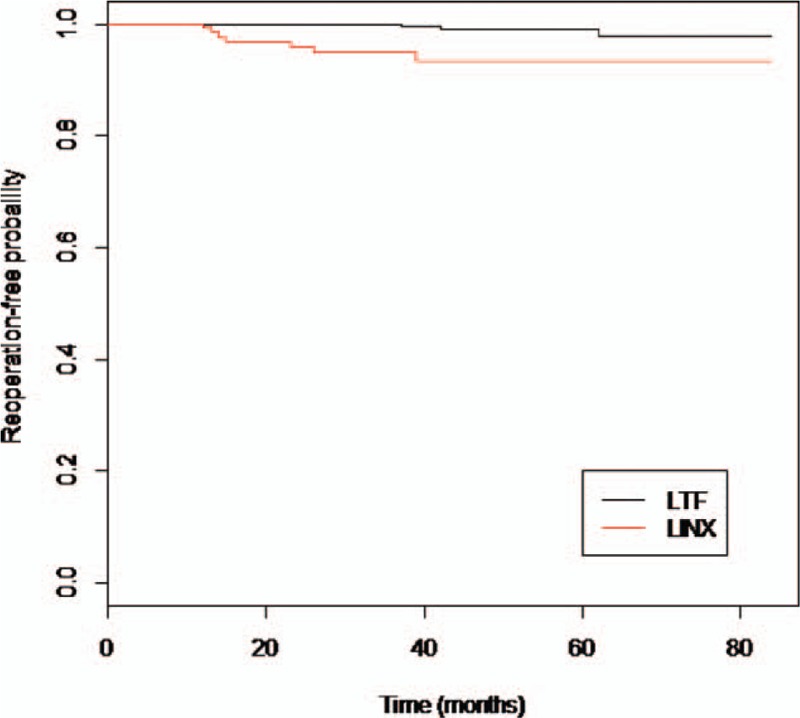
Reoperation-free probability in patients with LTF and LINX (stratified log-rank test for comparison of Kaplan–Meier curves, *P* = 0.556). LTF = laparoscopic Toupet fundoplication.

As indicated by the time–treatment interaction term in the GEE model, over time there was no statistical difference in PPI use (OR 1.18, CI 0.81–1.70; *P* = 0.388), gas-related symptoms (OR 0.69, CI 0.21–2.28; *P* = 0.532), and dysphagia (OR 0.62, CI 0.26–1.30; *P* = 0.241) between the LTF and LINX groups. As expected, the prevalence of dysphagia was significantly greater in patients with LINX at 3-month follow up (OR 9.42, CI 2.22–20.10; *P* < 0.001). At 1-year follow-up, there was no difference in the prevalence of dysphagia (interaction time term not significant, treatment term 0.78, CI −0.02 to 1.57; *P* = 0.357). There was no statistical difference in the reoperation-free probability between patients with LTF and LINX (stratified log-rank test, *P* = 0.556, HR 0.77, CI 0.234–2.57; *P* = 0.687). At 80 months, the estimated Kaplan–Meier reoperation-free probability survival was 0.97 (CI 0.88–0.99) in patients with LTF and 0.94 (CI 0.89–0.98) in patients with LINX (Fig. 3).

All study outcomes are summarized in Table 4.

**Table 4:**
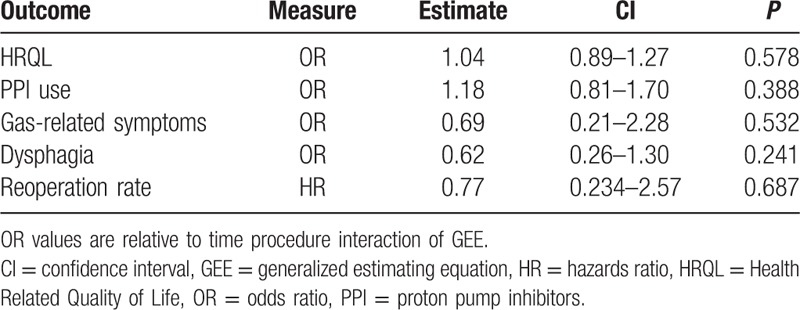
Results of primary and secondary study outcomes over time.

## 4 Discussion

The main finding of this observational study is that long-term health-related quality of life was similar in patients undergoing LTF and LINX. We also found that over time the GERD-HRQL scores had a similar decreasing trend in both groups. Longitudinal data, analyzed with appropriate statistical techniques such as GEE, can depict the temporal evolution of the outcome and take into account intra-individual variability. Since patient perception and satisfaction are reasonable and measurable indicators of the success of a surgical procedure, we assume that longitudinal quality of life data play an important role in the process of decision-making and counseling patients with GERD who are candidates for a laparoscopic antireflux procedure.

The long-held dogma that Nissen fundoplication is the ideal antireflux operation has recently been challenged. Systematic review and meta-analyses of randomized controlled studies have shown that LTF is equally effective to improve quality of life and is associated with less postoperative dysphagia and gas-related symptoms compared to Nissen fundoplication.^[19,20]^

Laparoscopic magnetic sphincter augmentation with the LINX device is an emerging surgical option for the treatment of GERD. A single-center study^[21]^ and a multicenter single-arm study,^[22]^ enrolling 100 patients each, evaluated the long-term results of magnetic augmentation and showed that the procedure provides significant and sustained control of reflux with minimal side-effects or complications up to 6 years of follow-up. However, no randomized trials exist that can validate the effectiveness of the LINX and reliably compare its results with other established surgical therapies. Interestingly, the patient profile for the LINX procedure is very similar to that required for the Toupet fundoplication, which has been successfully employed in patients with “mild” GERD.^[5]^ Three recent observational studies have compared LINX and laparoscopic Nissen fundoplication. Louie et al^[23]^ performed a retrospective case–control study comparing 66 patients undergoing LINX or Nissen fundoplication; at a mean follow-up of 6 and 10 months, respectively, scores on the GERD-HRQL scale significantly improved in both groups. Reynolds et al^[24]^ conducted a retrospective analysis of 1-year outcomes of 100 patients matched by PS; although the GERD-HRQL scores were similar in both groups, there were 10.6% of patients in the Nissen group complaining of severe gas-bloat symptoms compared with 0% in the LINX group. Finally, a smaller case-matched study by Sheu et al^[25]^ including 24 patients followed for an average of 7 months showed that severe dysphagia requiring endoscopic dilation was more frequent in the LINX group.

In the present study, for the first time, the long-term outcomes of a sizeable number of patients with LTF and LINX having a minimum 1-year follow-up and sharing similar characteristics of disease severity were compared in a longitudinal manner. We confirm that these surgical procedures equally normalize GERD-HRQL scores, and that the results are maintained over time for up to 7 years with a similar trend in both groups. As expected, dysphagia occurred more frequently at 3 months in patients undergoing the LINX procedure but this difference disappeared at 1 year. The reason for reoperation among patients with LINX was persistent dysphagia in 3, recurrent heartburn/regurgitation in 3, and erosion in 1. In all these individuals, the LINX device was safely removed through laparoscopy and a standard fundoplication was performed. Conversely, all 4 patients with LTF who required a reoperation complained of recurrent heartburn/regurgitation and underwent Nissen fundoplication.

A strength of this study is that we used a longitudinal model to analyze health-related quality of life scores over time. To our knowledge, this is the first time that the GERD-HRQL questionnaire is employed in a longitudinal study using beta GEE analysis. As opposed to pure cross-sectional studies, longitudinal studies with repeated measurements taken over time are more reliable in establishing causality.^[26]^ Waiting for a randomized clinical trial, the present study may provide some indications to surgeons and patients for selecting the most suitable laparoscopic antireflux procedure. The study sample size was sufficient to produce a narrow OR CI that did not encompass the clinical significance. The possible residual selection bias of our propensity-matched analysis was further mitigated by the fact that we have compared LTF to the LINX and not to the Nissen, a procedure that we usually reserve to patients with advanced GERD. In addition, we used similar criteria of exclusion from the study for both procedures.

Limitations of this study are that the GERD-HRQL is a validated, but still subjective test, and the LINX procedures were not standardized regarding crural repair. Hidden bias typical of an observational study cannot be excluded due to unmeasured and unmeasurable confounding factors. The PS model could be biased, and we did not consider possible measurable time-dependant confounders. Despite the sensitivity analysis showed negligible residual bias, we need to be cautious in interpreting the overall study results.

## 5 Conclusions

Both LTF and LINX can be safely offered as a first choice surgical option in patients with early stage GERD. However, a randomized clinical trial would be required to demonstrate the equivalence of the 2 procedures. Compared to fundoplication, LINX appears to be a simple, standardized, and easily reversible procedure that does not alter gastric anatomy; operative time is shorter but the cost of the device should be considered. Further research is needed to investigate correlation between longitudinal quality of life data with objective long-term outcome of these procedures.
